# Replicability and Heterogeneity of Awake Unrestrained Canine fMRI Responses

**DOI:** 10.1371/journal.pone.0081698

**Published:** 2013-12-04

**Authors:** Gregory S. Berns, Andrew Brooks, Mark Spivak

**Affiliations:** 1 Center for Neuropolicy, Emory University, Atlanta, Georgia, United States of America; 2 Comprehensive Pet Therapy, Sandy Springs, Georgia, United States of America; Ghent University, Belgium

## Abstract

Previously, we demonstrated the possibility of fMRI in two awake and unrestrained dogs. Here, we determined the replicability and heterogeneity of these results in an additional 11 dogs for a total of 13 subjects. Based on an anatomically placed region-of-interest, we compared the caudate response to a hand signal indicating the imminent availability of a food reward to a hand signal indicating no reward. 8 of 13 dogs had a positive differential caudate response to the signal indicating reward. The mean differential caudate response was 0.09%, which was similar to a comparable human study. These results show that canine fMRI is reliable and can be done with minimal stress to the dogs.

## Introduction

As the oldest domesticated species, the minds of dogs inevitably have been shaped by millennia of contact with humans [1], [2]. As a result of this physical and social evolution, dogs, more than any other species, have acquired the ability to understand and communicate with humans. Previously, our group published the first demonstration of fMRI in two awake, unrestrained dogs [3]. Using positive reinforcement, we trained the dogs to be highly cooperative during fMRI.

In our initial experiment, we used a simple instrumental conditioning task in which the required behavior was to place the head on a custom, anatomically designed chin rest and not move. After a variable interval of approximately 5 s, either of two separate hand signals was given that indicated either the presence or absence of a food reward that would be received. The left hand up indicated a hot dog reward, while both hands pointing toward each other horizontally indicated no reward. The hand signals were chosen to be easily distinguishable and were maintained for approximately 10 s. Consistent with the reward-prediction-error (RPE) theory of dopamine release [4], [5], we observed significant activation in the ventral caudate of both dogs in response to the hand signal that indicated “reward” relative to the hand signal that indicated “no reward” [3].

The main challenge of fMRI in dogs comes from subject motion. Historically, the usual approach has been to either anesthetize the animal [6], [7] or, as in rats and monkeys, immobilize them [8]-[13]. Clearly, if we wish to understand canine cognition, anesthesia is not an option. Moreover, anesthesia alters the hemodynamic response function [14] and may obliterate it in subcortical structures [15]. Immobilization is technically possible, although ethically objectionable for a dog and imposes unnatural emotional conditions that may bias the accuracy of a research study. Furthermore, as we have previously demonstrated, immobilization is unnecessary to acquire useful fMRI data. Instead, because dogs so readily follow human commands, they can be trained to cooperatively enter an MRI scanner and without restraint hold their head stationary so that we can conduct effective fMRI studies.

The goal of the current study was twofold: 1) determine whether our previous results could be replicated; and 2) determine the heterogeneity of canine caudate responses to human hand signals indicating the presence and absence of reward. Here, we report results in an additional nine dogs and improvements in training, image acquisition, and analysis of awake unrestrained canine fMRI data.

## Methods

### Ethics Statement

This study was performed in strict accordance with the recommendations in the Guide for the Care and Use of Laboratory Animals of the National Institutes of Health. The study was approved by the Emory University IACUC (Protocol # DAR-2001274-120814BA). All dogs' owners gave written consent for participation in the study.

### Training

Based on our initial experience, we have developed a training program for the dogs that teaches them to cooperatively enter the MRI scanner. The program is based on acclimatization to the MRI scanner noise, tight scanner enclosure, scanner steps, and operating vibrations and the shaping and ultimate chaining of several requisite behaviors. To do this, we constructed two replica MRIs, each of which consist of a tube of approximately the same dimensions as the inner bore of the actual Siemens MRI, a patient table, portable steps, and multiple simulated receiver coils that adhere closely to the dimensions of a human neck coil (see below). We also constructed a proprietary chin rest that facilitated comfort and proper positioning for the animals and that adapted the apparatus for the uniqueness of the canine anatomy. Once the animals became confident and competent regarding all the preparatory steps – proven by completing a simulated MRI in the replica apparatus – we then performed live scans in the actual Siemens MRI.

We compiled digitized audio recordings of the various scanner sequences. To aid in the necessary desensitization and acclimation to the scanner noises, when training, we played the recordings through a P.A. system aimed toward the simulator. We verified sound pressure levels with a handheld decibel meter to confirm that upon completion of the training process we approached the 96 dB level of the actual scanner. We located MRI simulators at the home of one of the owners and at a contracted training facility. We provided mock receiver coils for all the participating owners to take home, which encouraged daily training, expedited goal accomplishment, and better enabled the dogs to become comfortable with a key component of the process in the environment that is most familiar to the dog.

Only positive reinforcement, in combination with behavioral shaping, conditioning and chaining, are used in the training process. First, dogs are trained to place their head and paws in the head coil. Next, they are trained to place their chin on a chin rest placed horizontally across the head coil and hold this position until a release signal. The length of the hold is gradually increased up to 30 s. When the dogs are able to do this consistently with no discernible head motion, they are next trained to do this wearing canine ear muffs, which are initially introduced to the animals apart from the coil simulator. Concurrent with the initial sequences of the training, recordings of the scanner noise are introduced at low volume. Once the animal becomes conditioned at a low volume, the volume is gradually increased. Recordings of the scanner noise are introduced at low volume while the dog remains stationary in the coil. Once the dog demonstrates relaxed behavior, the volume is gradually increased. When the dogs are comfortable wearing the ear muffs in the head coil with the scanner noise of approximately 90 dB, they are then trained to go into the MRI tube which is placed on the floor. Subsequently, the simulated head coil is placed inside the tube. After the dog is consistently holding its head still in this configuration, the entire apparatus is raised on a table to the height of the actual scanner patient table. The dogs are trained to walk up steps into the tube. Finally, we increase the distance that the dog works away from the handler.

**Figure 1:**
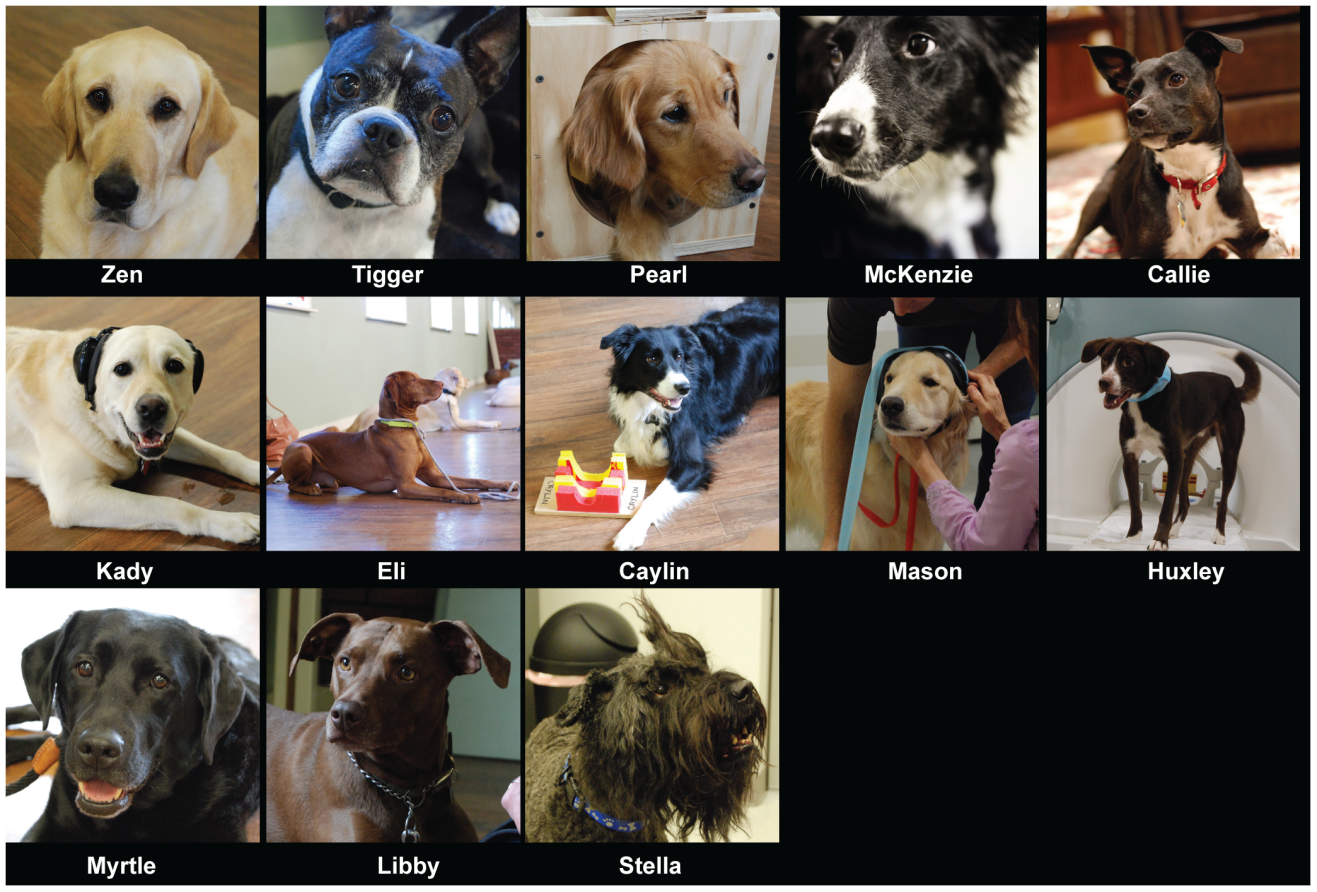
Dog participants. Pearl demonstrates the training device. Kady demonstrates the ear muffs. Caylin is with her chin rest, and Mason is getting his ear muffs wrapped to hold them in place.

With careful subject selection, some dogs can complete the training in as little as a few weeks. More commonly, we have found that 2-3 months of training with supervised practice sessions every other week leads to a high success rate on the first scan session. To date, we have trained 15 dogs, and 13 of 15 (87%) have successfully completed the scan (Table 1 and Fig. 1). Except for the first dog (Callie), whose scanning was accomplished by trial-and-error, ten of the other 12 dogs completed the scan on the first attempt. The remaining dogs became sensitized to the noise and required further desensitization. Both succeeded on the second attempt.

**Table 1:** Demographics of dogs.

Dog	Breed	Sex	Age (yrs)	Weight (lbs)	Special Skills
Zen	Yellow Lab	Male – neutered	3	70	Service dog training
Tigger	Boston Terrier	Male – neutered	6	26	Therapy dog
Pearl	Golden Retriever	Female - spayed	3	50	Service dog training
McKenzie	Border Collie	Female - spayed	4	35	Agility training
Callie	Feist	Female - spayed	3	25	Rescue. Hunts small animals
Kady	Yellow Lab	Female - spayed	2	52	Service dog training
Eli	Viszla	Male – intact	4	60	Gundog
Caylin	Border Collie	Female - spayed	4	44	Agility training
Mason	Golden Retriever	Male – neutered	8	67	Service dog
Huxley	Lab mix	Male – neutered	2	40	Rescue
Myrtle	Black Lab	Female –spayed	7	55	Service dog training
Stella	Bouvier	Female –spayed	5	65	Basic obedience
Libby	Pit mix	Female – spayed	7	50	Rescue. Basic obedience

### MRI Scanning

**Figure 2:**
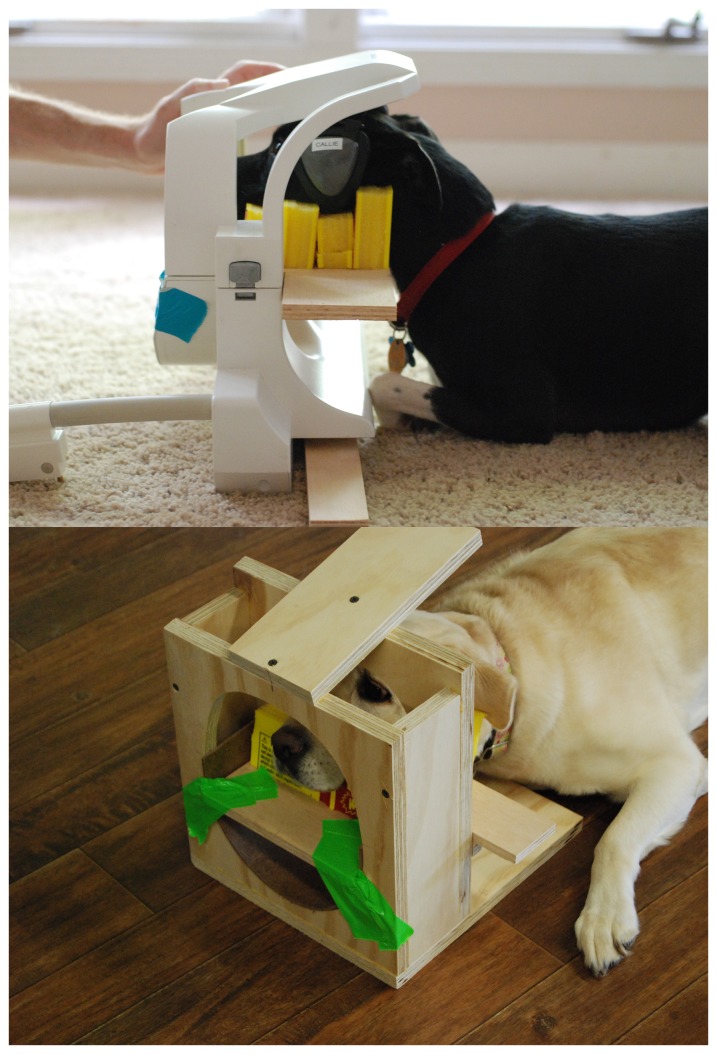
Neck coil configuration for canine imaging. *Above*: Callie demonstrates the chin rest inside the neck coil. Note the proximity of the upper element to the brain. *Below*: Kady demonstrates the chin rest placed within the training device that simulates the neck coil.

All scanning was performed on a Siemens 3T Trio whole-body scanner. Instead of the birdcage head coil used in our previous study, we have found that using a standard neck coil places the active element closer to the dog's brain (Fig. 2). Although less homogeneous in coverage than the birdcage, the upper element is in close proximity to the dog's brain, which provides a superior signal-to-noise ratio (SNR) of the brain in comparison to the birdcage coil, especially at the dorsal part of the brain (SNR ∼40 vs. 17 with birdcage.) More importantly, because the dog's shoulders and body are outside of the coil, we are less constrained by subject size. We can accommodate larger heads by simply lowering the chin rest.

The chin rest is constructed from firm foam boards, which are glued together to form a stack. Semicircles are cutout to match the shape of the dog's muzzle from just the nose to the ramus of the mandible. For training purposes, a plywood mockup of the neck coil is constructed for each dog. We then insert the dog's custom chin rest within the inner diameter of the coil (Fig. 2).

When performing an actual scan, immediately prior to the scan, we play audio recordings of the pertinent scan sequence through the scanner room speaker. As the dog settles in the scanner, we increase the recorded volume to match the decibel level of the actual scanner noise. While playing a continuous loop of the recording, once the sound level match we then begin the actual scan. The recordings are very effective at minimizing the startle response that would otherwise result from the sudden onset of the real scan. Once the scan actual begins, we turn off the scanner recording.

First, a single sagittal plane image is acquired as a localizer, which lasts 3 s (SPGR sequence, slice thickness = 4 mm, TR = 9.2 ms, TE = 4.16 ms, flip angle = 40°, 256×256 matrix, FOV = 220 mm). The localizer sound tends to be the most startling and unpleasant for the dogs. This is minimized by acquiring a single plane. Because the chin rest centers the dog in the left-right direction, a single sagittal image is all that is necessary for planning the field-of-view for the subsequent scans. For functional scans, we used single-shot echo-planar imaging (EPI) to acquire volumes of 25 sequential 3 mm slices with a 10% gap (TE = 28 ms, TR = 1400 ms, flip angle = 70°, 64×64 matrix, FOV = 192 mm). (The initial two dogs, Callie and McKenzie, were scanned in the birdcage coil with 28 slices and a TR = 1610 ms. Huxley was also scanned in the birdcage due to training preference.) Slices are oriented dorsally to the dog's brain (coronal to the magnet because the dog is positioned 90° from the usual human orientation) with the phase-encoding direction right-to-left. Sequential scans are preferred to minimize between-plane offsets when the dog moves. The 10% slice gap minimizes crosstalk for sequential acquisitions. The right-left phase encoding minimizes ghost images from the neck that would otherwise overlap into the dog's brain. TR is as short as possible to acquire enough slices to cover the entire brain of most dogs while not so short as to significantly decrease signal. Although perhaps not as significant for fMRI, the flip angle was still chosen to match the Ernst angle for gray matter [16]. For each dog, two runs of approximately 400 volumes were acquired, each lasting about 10 minutes.

**Figure 3:**
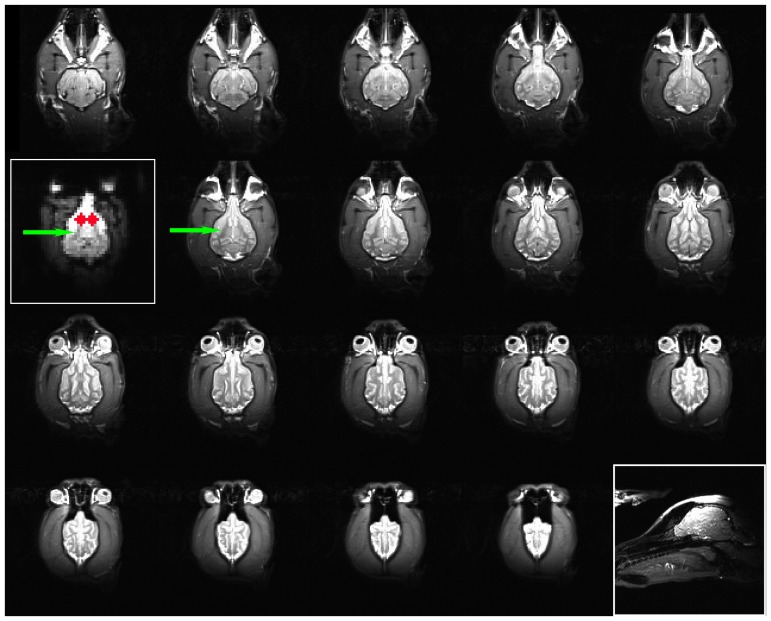
Structural image of Pearl with EPI comparison (*left inset*). A typical T2-weighted structural image with in-plane resolution of 1.5 mm (*lower inset*: localizer.) Because the phase-encoding direction is right to left, slight motion artifacts (e.g. eye movement) appear in that direction. The caudate is easily identified in the 2^nd^ row, inferior and posterior to the corpus callosum. The EPI image is from the mean of the functionals after motion correction and censoring. The slice shown contains the ventral part of the caudate and corresponds to the adjacent structural image. The internal capsule appears as a “chevron” (*green arrow*), and two ROIs for the caudate were placed anterior to that but posterior to the olfactory peduncle (*red*).

After the functional runs, a T2-weighted structural image was acquired with a turbo spin-echo sequence (25 2 mm slices, TR = 3940 ms, TE = 8.9 ms, flip angle = 131°, 26 echo trains, 128×128 matrix, FOV = 192 mm), which lasted 24 s. This sequence was optimized to yield good contrast between gray and white matter in the fastest time possible (Fig. 3). Importantly, it should be noted that because of the low weight of some dogs, the structural sequence can exceed the FDA SAR limit for humans. Although there is no SAR limit for dogs, we assume the same limit as if they were humans (4 W/kg averaged over the whole body for any 15 minute period or 3 W/kg over the head for a 10-minute period). Decreasing the flip angle is an effective way to decrease SAR. In our cohort, SAR is typically 1.5-2 W/kg, but has been as high as 3.97 W/kg in the smallest dog. Even at that level, the scan is only 24 s long which means a negligible rise in tissue temperature.

### Event recording

Trial events were recorded by an observer via a four-button MRI-compatible button-box. These events included hand signal onset, offset, and reward. A laptop running Matlab (MathWorks) and Cogent (FIL, University College London) was connected via serial port to the button box, and recorded both the button-box responses by the observer, as well as the scanner sequence pulses.

### Task

We used a simple instrumental conditioning task in which the required behavior was to place the head on the chin rest and not move. After a variable interval of approximately 5 s, a hand signal was given that indicated the presence or absence of a food reward that would be received. The left hand up indicated a food reward (a small piece of sausage), while both hands pointing toward each other horizontally indicated no reward. The hand signals were chosen to be easily distinguishable and were maintained for approximately 10 s. The dog had to continue holding still during this period. Dogs had been amply trained on these hand signals in the simulator prior to the final scan session. Because the dogs had been trained to go into the head coil in a “sphinx” position, the handler gave the hand signals from the head end of the scanner, facing the dog. We performed 20 repetitions of each trial type (total of 40 trials), split evenly between two functional runs. Trial types were random in order. To ensure that the dogs paid attention to the onset of the hand signal, the reward hand signal was maintained until the food was actually delivered. This prevented the dogs from associating the offset of the signal with the food. The food reward was always held in the right hand, and it was delivered directly to the dog's mouth by reaching into the bore. The food consumption resulted in head movement, but the dogs were trained to replace their head in the chin rest and await the next hand signal. The next hand signal began approximately 5 s after the dog had replaced its head in the chin rest. This interval allowed for several TRs to restabilize the MR signal. Scans during the food consumption period were typically discarded from analysis (see below).

### Preprocessing and analysis

All functional data was pre-processed using AFNI and its associated functions. DICOM files of the EPI runs were first converted to AFNI BRIK format using the to3d command. The EPI runs were then subjected to motion correction using 3dvolreg's 6-parameter affine transformation, employing a two-pass method, where the first-pass results in a crude alignment and the second-pass a fine alignment. All volumes were aligned to a reference volume, which was either the first volume of the first run, or a manually chosen volume from the first run based on a visual inspection.

Three separate methods were used to censor volumes with remaining motion artifacts. First, 3dToutcount was used to output the fraction of outlier voxels for each volume. 3dToutcount defines outliers as those voxels whose signal intensity deviates from the median absolute deviation of the time series. Volumes with a fraction larger than a cut off (0.1 or 0.001, depending on the dog) were censored from the statistical analysis. Second, 1d_tool.py was used to censor volumes based on the amount of estimated motion outputted from 3dvolreg. 1d_tool.py computes the derivative of the time series by subtracting from each volume the preceding volume, as well as the Euclidean norm of the rotation and translation parameters outputted from 3dvolreg. We used a Euclidean norm cut-off that varied between 1 and 1.6, depending on the subject, to generate the censor file. Finally, we visually inspected the resulting time series with the censored volumes from 3dToutcount and 1d_tool.py, and censored any volumes that showed obvious artifacts. On average, 43% of the total EPI volumes were retained for each subject (ranging from 30% - 59%).

The EPI images were then smoothed and normalized to %-signal change. Smoothing was applied using 3dmerge, with a 6 mm kernel at Full-Width Half-Maximum (FWHM). The size of the smoothing kernel was chosen based on the anticipated size of the striatal response to the hand signal predicting reward. To convert signal intensity values to %-signal change, 3dcalc was used to subtract and then divide by the mean EPI image (generated from the 3dTstat –mean option). These values were then converted to percentages by multiplying by 100. These resulting scaled EPI images were then inputted into the General Linear Model.

For each subject, a General Linear Model was estimated for each voxel using 3dDeconvolve. The task-related regressors in this model included, 1) reward hand signal, 2) no reward hand signal, and 3) reward consumption. All three task-related regressors were impulse functions – that is, their duration was not modeled. In our original experiment, we observed that the hemodynamic response function (HRF) in the caudate peaked 3-6 s after the onset of the hand signal, which is similar to the human HRF in the caudate. Based on this, all events were convolved with a single gamma-function. We used the GAM function in AFNI's 3ddeconvolve with default parameters, which results in an HRF that peaks at 5 s. To help control for subject movement, 6 motion regressors outputted from 3dvolreg were also included in the model. To account for differences between runs, a constant and linear drift term was included for each run.

Because the heterogeneity in the canine brain shape and size precluded group normalization, we performed an individual-based ROI-analysis. For each dog, two spherical ROIs (radius 6 mm) were located anatomically on the mean EPI image and corresponded to the left and right ventral caudate. Although the caudate is not clearly visible on EPI images, we can visually approximate its location anterior to the “chevron” created by the internal capsule and posterior to the olfactory bulb and by reference to the dog's structural image (Fig. 3). The average difference in response to the reward hand signal versus no-reward hand signal was then calculated from these ROIs. The ROI values were analyzed with a mixed-effect model in SPSS 21 (IBM). This model included fixed effects for hand signal (reward, no reward) and side (left, right), signal x side as a repeated effect, and dog as a random effect.

## Results

When contrasting the hand signal for reward to the hand signal for no reward, 8 out of 13 dogs (62%) demonstrated a positive differential signal in the caudate (averaged over left and right). However, one dog – Caylin – was a negative outlier based on Grubbs' test (Z = 2.37) and was subsequently excluded from further analyses. With the mixed-effect model for the remaining dogs, hand signal was significant [F(1,36) = 4.81, p = 0.035] but side was not [F(1,36) = 0.01, p = 0.940]. Pairwise comparison of reward vs. no reward signal showed a mean difference of 0.093% (se 0.042%), indicating a positive effect consistent with our original study.

**Figure 4:**
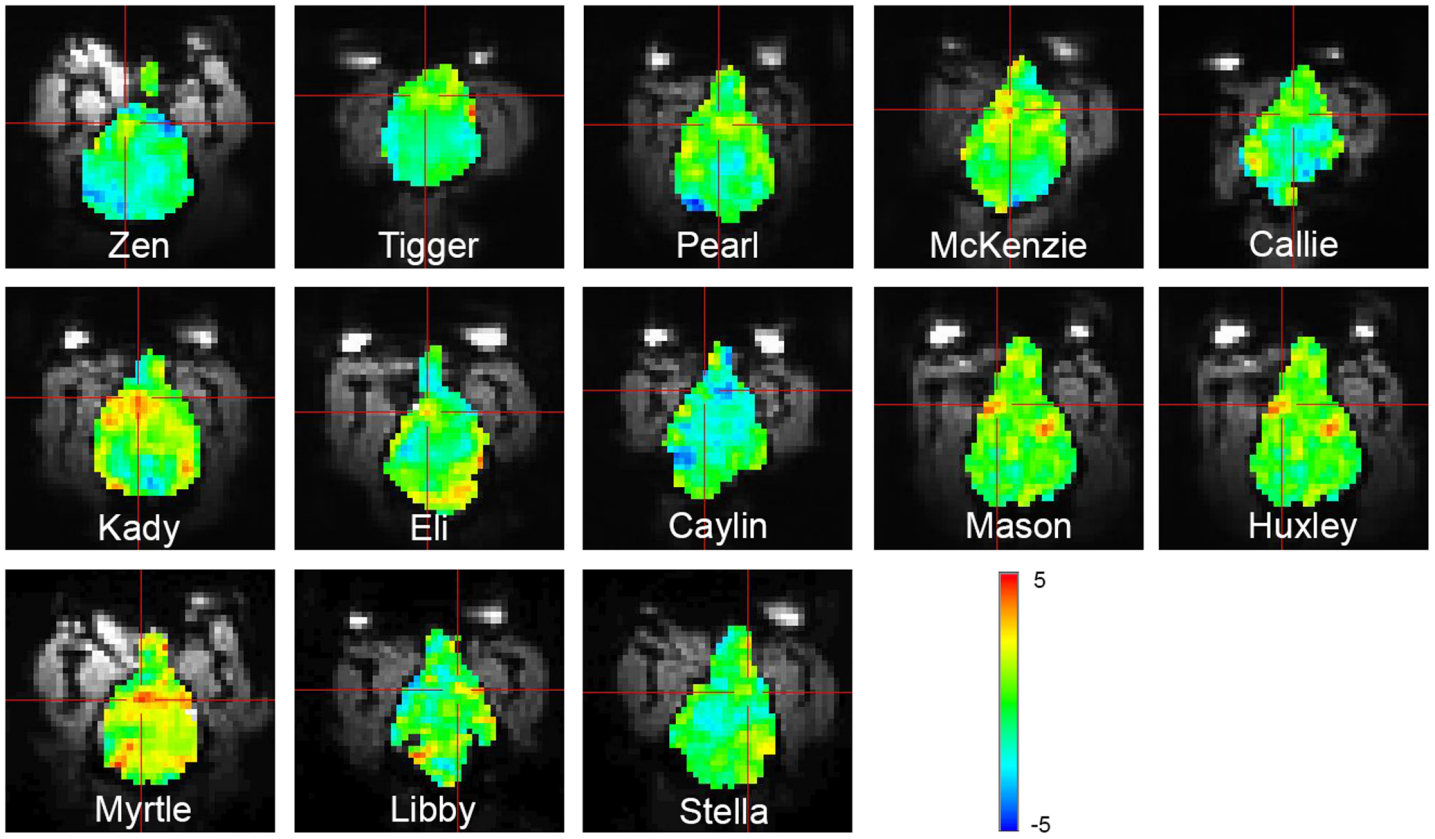
Unthresholded t-maps of hand signal for “reward” vs. hand signal for “no reward.” The slice containing the ventral caudate for each dog is shown with the crosshairs over the area of maximal activation in the vicinity of the caudate. Colorbar indicates t-values.

**Figure 5:**
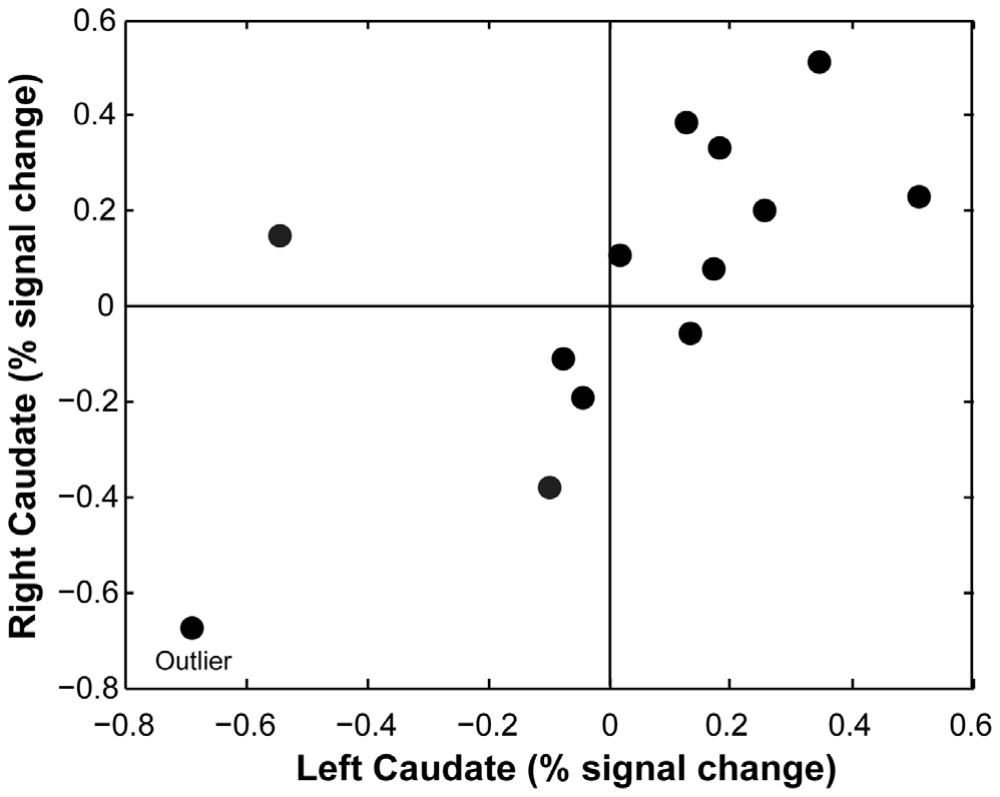
Comparision of left and right caudate activation. The differential activity between “reward” hand signal and “no reward” hand signal was generally similar in both the left and right caudate for each dog. One dog (Caylin) was an outlier with deactivation bilaterally. 7 of 12 dogs had positive activations bilaterally, and 9 of 12 dogs had positive activations on at least one side.

The subjectwise heterogeneity of these results is just as interesting as the replicability. Similar to human studies, the whole-brain activation showed varying patterns and intensity of activity across the cortex in each of the subjects (Fig. 4). But within the caudate ROIs, there were no significant differences in left/right activation. In fact, most dogs had similar levels of activation on both sides (Fig. 5). One dog had a positive differential activation on the right and negative on the left, which, when both sides were averaged together resulted in an apparent “deactivation” to the reward hand signal. This seemed to be the pattern in the few dogs that had apparent deactivations, with the average value being driven negative by either the left or right caudate, with the other side being positive or close to zero.

**Figure 6:**
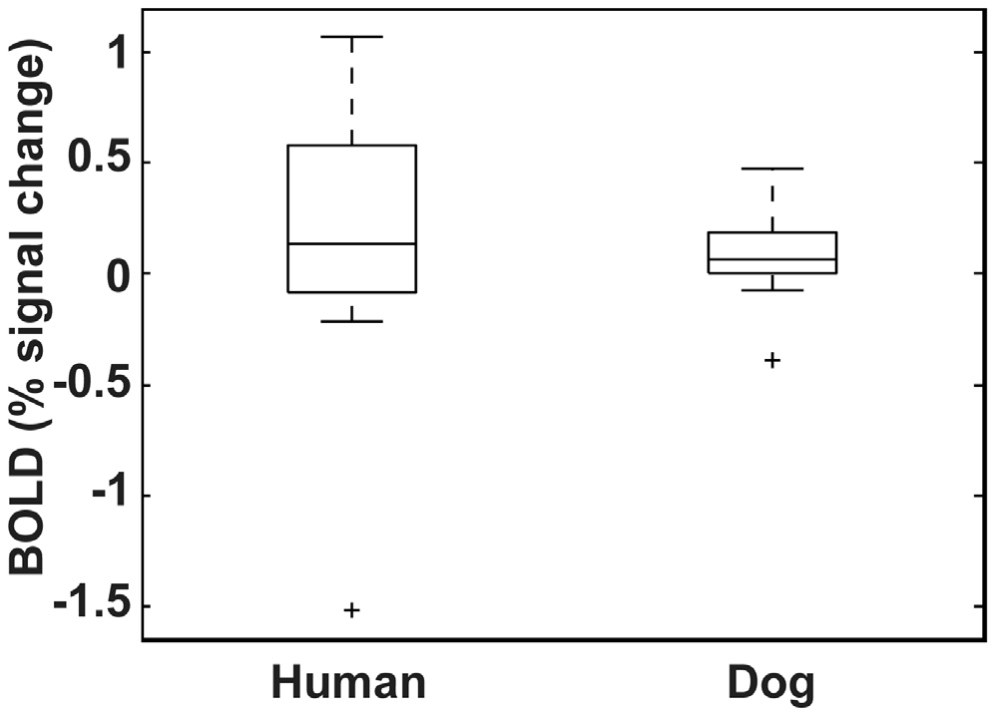
Comparison of caudate response in humans and dogs. The human data is from an instrumental conditioning task. The caudate response is to a visual cue indicating the imminent receipt of fruit juice, to which participants had to press a button to receive the juice [17]. The canine data is in response to the hand signal indicating “reward.” For comparison to each other, both dog and human activations are referenced to an implicit baseline.

Although we did not find evidence for strong laterality within the caudate, there was still substantial heterogeneity in the overall level of activation in the caudate. As a point of reference, we compared the level of activation and heterogeneity of the dogs to a similar study we had conducted in humans [17]. In this experiment, human volunteers (N = 17) were instructed to press a button to a colored circle on a computer monitor. Pressing the button delivered a squirt of fruit juice into the participant's mouth 4 s later. On ‘catch’ trials, the juice was delivered at 8 s. Consistent with a reward-prediction error (RPE) model, we observed a significant striatal response to the cue in the regular trials. The form of this trial closely paralleled the dogs' task, which was to stay in the chin rest and await the delivery of a treat. Caudate ROIs were placed anatomically in the human study, and we fit the same gamma function to the human data as the dog data and estimated the subjectwise coefficients to the cue (Fig. 6). The median caudate activation of the dogs was 0.06% while in humans it was 0.14%. The 25^th^ percentile was approximately zero, and both samples had one negative outlier. Interestingly, the dogs had a smaller range of caudate activations than the humans. The number of humans with a positive caudate response was 11 of 17 (65%) compared to 9 of 12 dogs (75%) with a positive caudate response to the hand signal indicating reward (i.e. not the differential).

To further understand the heterogeneity of the dog caudate response, we conducted an exploratory analysis by including subjectwise covariates in the mixed-effect model. One potential variable that was correlated with caudate activity was whether the dog was bred or trained to be a service / therapy dog. Removing side from the model because it was not significant, and including a dummy variable for service dog, we found that service dogs had greater caudate activation than non-service dogs [F(1,12) = 4.1, p = 0.066].

## Discussion

In this study, we replicated the results of our initial report of caudate activation in awake unrestrained dogs [3]. In fact, in comparison to an analogous human data set, the caudate activation in the dogs may be less variable. This demonstrates that fMRI in awake unrestrained dogs is not only possible, but that it is reliable. We assume that the replicability has as much to do with the training procedure as the fMRI signal: reliable activations can be achieved with consistent training. This can be a challenge when using community volunteers, who may not consistently practice at home. In our protocol, we rely on weekly or biweekly classes in a structured training environment to assess both the dogs' and the owners' progress. We also use these classes to tailor the training program for the needs of a particular dog when the dog has demonstrated difficulty mastering a specific element of the ultimate task (e.g. entering the tube or walking up the steps).

Because the dogs were lying motionless in the MRI, the task may have the appearance of being entirely passive. The extensive training procedure suggests otherwise. Throughout the training, the dogs learned to assume a “down-stay” position in the head coil and that they would only be rewarded for maintaining this position without moving. Thus, while the task may appear to be pure classical conditioning, we believe that it is actually an instrumental learning task. This is important in reference to the comparison human study, which was an instrumental task because the human participants had to press a button to receive their reward. In subsequent human studies we have found that “working” for a reward is associated with greater striatal activity than purely passive tasks [18]. Assuming that the dogs were, in fact, working, this may explain the robustness and replicability of their caudate activation. Although we do not have an extrinsic measure of work, our results raise the interesting possibility of using the relative caudate activation as an intrinsic measure of motivation.

In the prior, albeit limited, literature on canine fMRI, there have been several inconsistencies. Some have reported delayed decreases in BOLD signal [7] while others have found increases [6]. However, these studies were done under anesthesia. In view of recent reports of the blunting effect of anesthesia on the BOLD signal in marmosets, especially in subcortical structures like the caudate [15], these inconsistencies may be a result of the effect of anesthesia and respiration. Moreover, it is impossible to study cognition under anesthesia. Thus, awake fMRI may not only result in a larger BOLD signal, but it is likely more reliable, and certainly more appropriate for cognitive studies.

Even though the canine caudate activations were replicated, there was still heterogeneity in these responses (although less than humans). There are several possible sources for this variation. FMRI signals, by their nature, are inherently noisy, and this could bias the apparent activation in any individual subject. Sufficient trial repetition should mitigate this source of variability, but the dogs cannot stay in the MRI as long as humans, and so the number of trials is less than most human studies. Moreover, this limitation could be compounded by the retention of less than 50% of scan volumes due to subject motion. Excluded volumes generally followed the administration of the reward when the dog moved his head while eating but not so much during the hand signal period. In any case, the censoring procedure was applied to the design matrix after convolution with the HRF so that even when volumes were discarded, the expected hemodynamic response was scaled to the appropriate point in time, even when intervening volumes were removed. The adequacy of the number of trial repetitions is a complex issue and depends on the effect size. While more repetitions are desirable to increase the SNR, too many repetitions may result in suppression of the fMRI response due to habituation and work against signal detection. 20 repetitions seemed a good compromise between signal detection and habituation without taxing the dogs' attention span.

Because the ROIs were placed anatomically, we expect some degree of mislocation, which will also contribute to the heterogeneity. Other sources of variability are the same as humans and include differences in subjective value of the reward, motivation, ability to learn associations, and stress in the MRI. Even though we focus on the caudate, individual dogs may use different perceptual and cognitive processes to evaluate the meaning and value of the hand signal. The dogs may also have different emotional and motivational states that contribute to their perception of the hand signals, or the value of the hot dog reward. Finally, because the signals are given by each dog's owner, there will be variability in how each person performs the task. Despite all the potential sources of variability, we are reassured by the consistency of the measured caudate responses. In both dogs and humans, the percentage of subjects with a positive caudate response was similar, ranging from 65-75%. Due to the sample size and the variability in regions activated, we cannot interpret the heterogeneity in cortical responses, but we assume that much of this variability is due to the aforementioned sources.

Notwithstanding these sources of variability, the observed heterogeneity of the caudate responses may represent real differences in both cognitive and motivational states of the individual dogs. First, the negative outlier deserves comment. Caylin is a border collie who is skilled in agility competition, and the handler relies heavily on hand signals in competition. A common signal for “stay” is to raise the hand. It is possible that the signal we used for reward was conflicted with a preexisting signal that Caylin had already learned. Second, the nominally greater caudate activation in service dogs raises the intriguing possibility that these dogs may find human signals and interactions instrinsically more rewarding than non-service dogs. If true, we do not know whether it is because of breeding or training, but this opens a new area of research in the future.

In conclusion, we find that caudate responses in awake unrestrained dogs during fMRI are reliable and consistent with reward-prediction error models. Moreover, the magnitude of the canine caudate response is similar to that of humans, while the between-subject variability in dogs may be less than humans. These results confirm the viability of awake unrestrained canine fMRI for study of canine cognition in an ethical, non-stressful manner.
