# Correction: Replicability and Heterogeneity of Awake Unrestrained Canine fMRI Responses

**DOI:** 10.1371/journal.pone.0098421

**Published:** 2014-05-16

**Authors:** The PLOS ONE Staff

There is an error in Figure 4. When the montage of images was assembled, the image of Mason was accidentally inserted for Huxley. The authors have provided a corrected version here.

**Figure 4:**
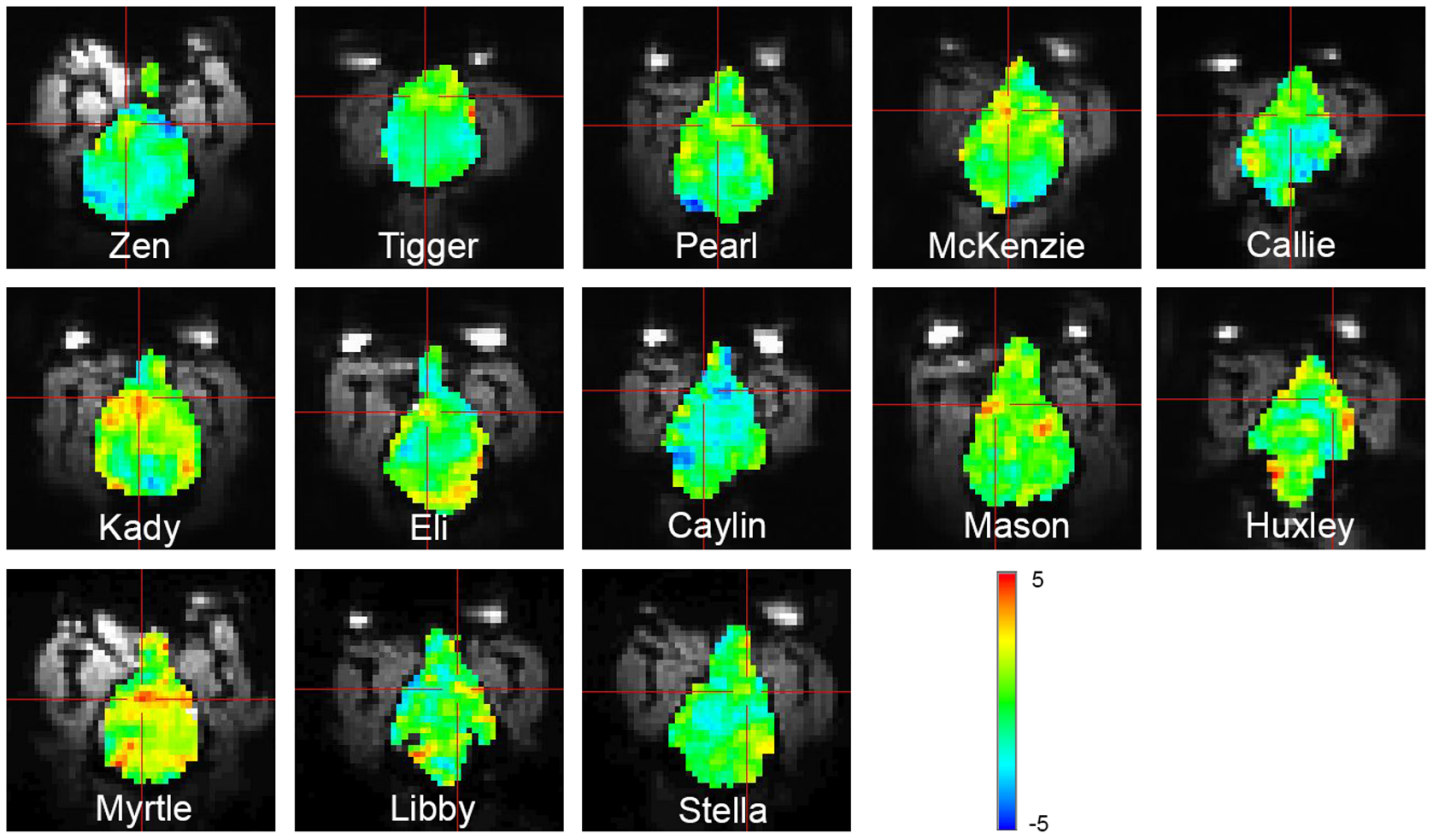
Unthresholded t-maps of hand signal for “reward” vs. hand signal for “no reward.” The slice containing the ventral caudate for each dog is shown with the crosshairs over the area of maximal activation in the vicinity of the caudate. Colorbar indicates t-values.
